# Imperatorin Positively Regulates Melanogenesis through Signaling Pathways Involving PKA/CREB, ERK, AKT, and GSK3β/β-Catenin

**DOI:** 10.3390/molecules27196512

**Published:** 2022-10-02

**Authors:** Taejin Kim, Chang-Gu Hyun

**Affiliations:** Jeju Inside Agency and Cosmetic Science Center, Department of Chemistry and Cosmetics, Jeju National University, Jeju 63243, Korea

**Keywords:** B16F10, imperatorin, isoimperatorin, melanogenesis, signaling pathway

## Abstract

The present study investigated the melanogenic effects of imperatorin and isoimperatorin and the underlying mechanisms of imperatorin using a mouse melanoma B16F10 model. Interestingly, treatment with 25 μM of either imperatorin or isoimperatorin, despite their structural differences, did not produce differences in melanin content and intracellular tyrosinase activity. Imperatorin also activated the expression of melanogenic enzymes, such as tyrosinase (TYR) and tyrosinase-related proteins TYRP-1 and TYRP-2. Mechanistically, imperatorin increases melanin synthesis through the cyclic adenosine monophosphate (cAMP)-dependent protein kinase (PKA)/cAMP-responsive element-binding protein (CREB)-dependent upregulation of microphthalmia-associated transcription factor (MITF), which is a key transcription factor in melanogenesis. Furthermore, imperatorin exerted melanogenic effects by downregulating extracellular signal-regulated kinase (ERK) and upregulating phosphatidylinositol 3 kinase (PI3K)/protein kinase B (AKT)/glycogen synthesis kinase-3β (GSK-3β). Moreover, imperatorin increased the content of β-catenin in the cell cytoplasm and nucleus by reducing the content of phosphorylated β-catenin (p-β-catenin). Finally, we tested the potential of imperatorin in topical application through primary human skin irritation tests. These tests were performed on the normal skin (upper back) of 31 volunteers to determine whether 25 or 50 µM of imperatorin had irritation or sensitization potential. During these tests, imperatorin did not induce any adverse reactions. Taken together, these findings suggest that the regulation of melanogenesis by imperatorin can be mediated by signaling pathways involving PKA/CREB, ERK, AKT, and GSK3β/β-catenin and that imperatorin could prevent the pathogenesis of pigmentation diseases when used as a topical agent.

## 1. Introduction

Melanin plays a crucial role in protecting the skin by absorbing and scattering ultraviolet light from the sun, neutralizing free radicals, and scavenging toxic drugs and chemicals. However, the underproduction of melanin and melanin deficiency can cause visible hypopigmentation of the epidermis, which may manifest as vitiligo, albinism, and Pityriasis alba [1]. In particular, vitiligo is a chronic skin problem that is characterized by patchy loss of skin pigmentation and affects about 1% of the world’s population. Melanin biosynthetic dysfunction due to the selective damage or apoptosis of melanocytes in the skin is the major cause of this disease [2]. Skin pigmentation relies not only on the proliferation and differentiation of melanocytes, which are pigment-producing cells, but also on the regulation of epigenetic genes that are mediated by other adjacent cell types, particularly dermal fibroblasts and epidermal keratinocytes [3]. Melanocytes that are located in the basal layer of the epidermis produce melanin in melanosomes and deliver it to keratinocytes. Melanogenesis occurs via complex signal transduction pathways involving a combination of enzymatic catalysis and a series of chemical reactions [4].

Tyrosinase (TYR) regulates the rate-limiting step in melanogenesis and oxidizes l-3,4-dihydroxyphenylalanine (L-DOPA) into l-dopaquinone. This step is common in both eumelanin and pheomelanin production. The subsequent steps involve two additional enzymes that are needed to produce eumelanin: tyrosinase-associated protein 1 (TYRP-1) and TYRP-2 [5]. Microphthalmia-associated transcription factor (MITF) is a key transcription factor that plays a vital role in melanogenesis via transcriptional regulation of TYR, TYRP-1 and TYRP-2 expression. Several signal transduction pathways that are involved in melanogenesis regulate MITF and TYR expression, including the MC1R/α-MSH, PI3K/AKT, MAPK, WNT/β-catenin, and NO signaling pathways [6]. Based on its regulatory role in melanogenesis, MITF has been proposed as a novel target for functional therapeutics that could provide sustained effects in modulating melanin production.

**Figure 1:**
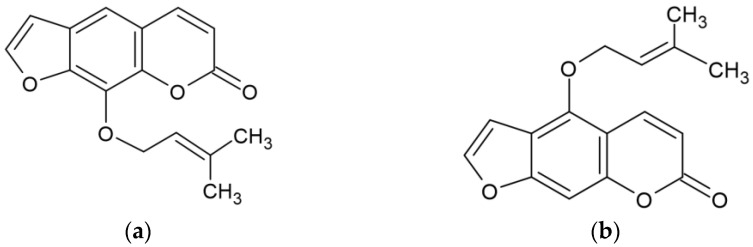
The structures of (**a**) imperatorin (CAS 482-44-0) and (**b**) isoimperatorin (482-45-1).

A continuous screening program aimed at discovering novel melanogenesis regulators using mouse B16F10 models uncovered some plant- and microbial-derived natural products that could play a vital role as melanogenic activators and inhibitors [7,8,9,10]. As an extension of those findings, we investigated the melanogenic effects of imperatorin and the underlying mechanisms using multiple approaches (Figure 1).

Imperatorin, known as ammidin, marmelide, and marmelosin, is a naturally occurring active furocoumarin and phytochemical that is mainly found in citrus fruits (such as *Citrus grandis* and *Citrus maxima*) and some species of the genus *Angelica* that are used as medicine (such as *Angelica biserrate*, *Angelica dahurica*, *Angelica archangelica*, *Angelica pubescens* and *Angelica albicans*). It is known to exert many pharmacological properties, including anti-inflammatory effects on inflammation-associated lung, liver, osteoarthritis, ulcerative colitis, and neuro diseases [11,12,13,14,15,16,17,18,19,20] as well as anticancer [21,22,23,24] and antiviral activities [25,26,27]. A recent study has shown that imperatorin IMP can ameliorate PS-induced learning and memory deficits through the BDNF/TrkB and ERK/CaMKIIα/CREB signaling pathways and the hypothalamic–pituitary–adrenal axis [28]. Imperatorin markedly inhibits activation of the PI3K/AKT/NFκB pathway by suppressing the phosphorylation of PI3K, AKT, and p65 in ectopic endometrium tissue [29]. Furthermore, imperatorin alleviates the metabolic and vascular alterations that are induced by high-fat/high-fructose diets (HFFDs) in rats through the restoration of adiponectin receptor 1 and eNOS expression and the suppression of p47phox expression [30]. However, it remains unknown as to whether imperatorin modulates melanogenesis by regulating the expression of MITF and TYR, including the MC1R/α-MSH signaling pathway, the PI3K/AKT signaling pathway, the MAPK signaling pathway, and the WNT/β-catenin signaling pathway. Therefore, the aim of this study was to evaluate the effects of imperatorin on melanogenesis and its molecular mechanisms in mouse melanoma B16F10 cells.

## 2. Results

### 2.1. Effects of Imperatorin and Isoimperatorin on the Viability, Melanin Content and Cellular Tyrosinase Activity of B16F10 Cells

**Figure 2:**
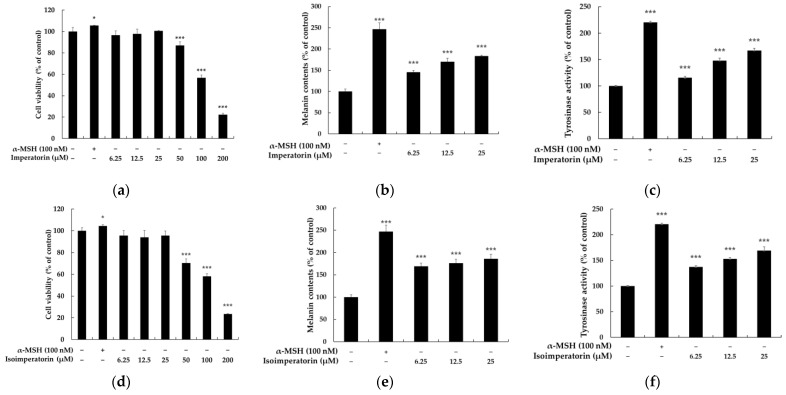
The effects of imperatorin and isoimperatorin on the viability (**a**,**d**), melanin production (**b**,**e**), and tyrosinase activity (**c**,**f**). Cells were plated in 24-well plates (1 × 10^4^ cells/well) for 24 h and then treated with imperatorin and isoimperatorin (200, 100, 50, 25, 12.5, and 6.25 μM) for 24 h. The cytotoxicities of imperatorin and isoimperatorin were evaluated using MTT assays. For the evaluation of melanin production and tyrosinase activity, cells were placed in 60 mm cell culture dishes (7.0 × 10^4^ cells/dish), incubated for 24 h, and then treated with imperatorin and isoimperatorin (25, 12.5 and 6.25 μM) for 72 h in the presence of α-MSH (100 nM). α-MSH treatment was used as the positive control. The results are presented as the mean ± SD from three independent experiments. * *p* < 0.05, *** *p* < 0.001 vs. the untreated control group.

We first aimed to evaluate the cytotoxic effects of imperatorin and isoimperatorin in B16F10 melanoma cells and to determine the maximum concentrations to apply in our subsequent experiments based on 3-(4,5-dimethylthiazol-2-yl)-2,5-diphenyltetrazolium bromide (MTT) cell viability assays. After treatment with various concentrations (6.25, 12.5, 25, 50, 100, and 200 μM) of imperatorin and isoimperatorin for 72 h, there were no significant differences in the viability of B16F10 cells for doses of imperatorin and isoimperatorin up to 25 μM (Figure 2a,b). Therefore, we used imperatorin and isoimperatorin at concentrations below 25 μM in subsequent experiments. To examine whether imperatorin and isoimperatorin affected melanogenesis of B16F10 cells, the cells were treated with imperatorin and isoimperatorin (6.25–25 μM) and α-MSH (100 nM) for 72 h. The α-MSH treatment was used as a positive control for melanogenesis. As shown in Figure 2c,d, 25 μM of imperatorin and isoimperatorin increased the melanin content to 183% and 185%, respectively. The cellular tyrosinase activity also significantly increased due to treatment with imperatorin and isoimperatorin compared to the untreated control group. Compared to the untreated control, 25 μM of imperatorin (Figure 2e) and isoimperatorin (Figure 2f) increased tyrosinase activity to 166% and 169%, respectively. These results were consistent with those that compared the effects of imperatorin and isoimperatorin on the melanin content of B16F10 cells. Both isoimperatorin and imperatorin showed similar increases in melanin content and tyrosinase activity. Therefore, further experiments were performed to evaluate the effects of imperatorin on melanogenesis.

### 2.2. Effects of Imperatorin on the Abundance of Melanogenic Enzymes and MITF

**Figure 3:**
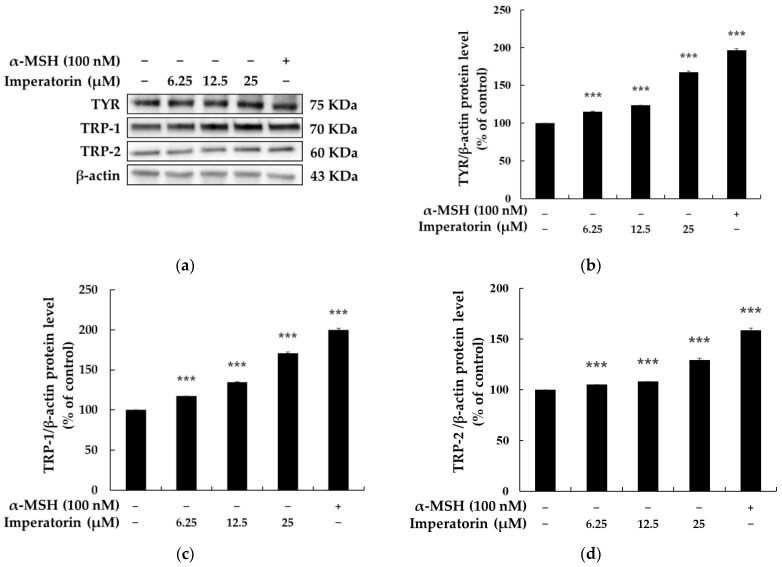
The effects on the protein expression of TYR, TYRP-1, and TYRP-2 in B16F10 melanoma cells when the cells were treated with imperatorin (25, 12.5, and 6.25 μM) for 48 h: (**a**) Western blotting results; protein levels of (**b**) TYR, (**c**) TYRP-1, and (**d**) TYRP-2. β-Actin was used as a loading control. The results are presented as the mean ± SD from three independent measurements using ImageJ software. *** *p* < 0.001 vs. the untreated control group.

**Figure 4:**
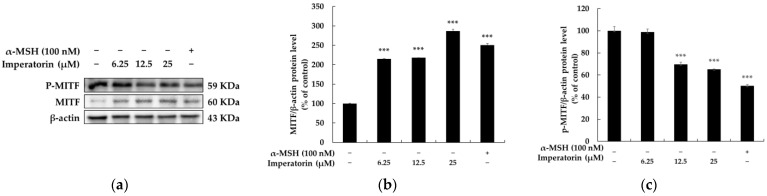
The effects on the protein expression and phosphorylation of MITF in B16F10 melanoma cells when the cells were treated with imperatorin (25, 12.5, and 6.25 μM) for 24 h: (**a**) Western blotting results; (**b**) protein levels of MITF; (**c**) phosphorylation level of MITF. β-Actin was used as a loading control. The results are presented as the mean ± SD from three independent measurements using ImageJ software. *** *p* < 0.001 vs. the untreated control group.

Since imperatorin positively and directly regulates melanin content and TYR enzymatic activity, we next examined whether imperatorin affects the abundance of melanogenic enzymes, including TYR, TYRP-1, and TYRP-2 proteins. Immunoblotting showed that imperatorin treatment upregulates the expression of TYR, TYRP-1, and TYRP-2 proteins in a concentration-dependent manner (Figure 3). MITF has been reported to act as a master transcription factor for the expression of the tyrosinases TYRP1 and TYRP2 during melanin biosynthesis. We next investigated whether imperatorin affects the abundance of MITF in B16F10 cells. Figure 4 shows that imperatorin upregulates MITF protein expression and downregulates MITF phosphorylation in a dose-dependent manner. When taken together, these results clearly suggest that imperatorin stimulates melanogenesis via the MITF-mediated upregulation of TYR, TYRP-1, and TYRP-2 at a cellular level.

### 2.3. Effects of Imperatorin on the PKA–CREB Signaling Pathway

**Figure 5:**
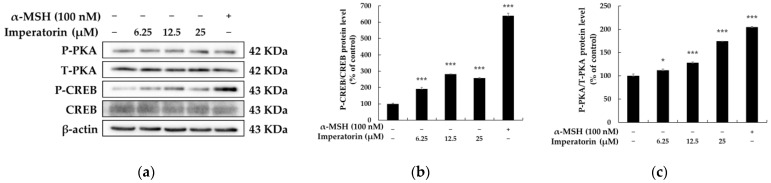
The effects on the protein expression of PKA and CRE in B16F10 melanoma cells when the cells were treated with imperatorin (25, 12.5 and 6.25 μM) for 15 min: (**a**) Western blotting results; protein levels of (**b**) P-CRE and (**c**) P-PKA. β-Actin was used as a loading control. The results are presented as the mean ± SD from three independent measurements using ImageJ software. * *p* < 0.05, and *** *p* < 0.001 vs. the untreated control group.

α-MSH, which is a hormone that is involved in the first stage of melanogenesis, stimulates phosphorylation of protein kinase A (PKA) and cyclic adenosine monophosphate (cAMP) response element-binding protein (CREB) in melanocytes, thus inducing the synthesis of the downregulating proteins that are associated with melanogenesis [31,32,33,34]. Therefore, we next examined whether imperatorin induces melanogenesis via the PKA/CREB signaling pathway in B16F10 cells. As a result, we confirmed that phosphorylation of PKA and CREB is significantly increased when B16F10 cells are stimulated with imperatorin in a dose-dependent manner (Figure 5). When taken together, these data show that the melanogenesis effects of imperatorin closely involve the PKA/CREB signaling pathway and occur after the upregulation of TYR, TYRP-1, and TYRP-2 via MITF.

### 2.4. Effects of Imperatorin on the MAPK Signaling Pathway

**Figure 6:**
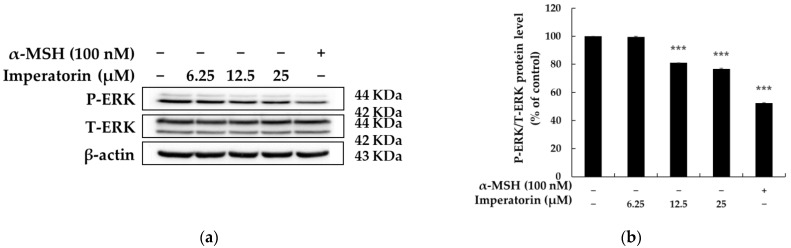
The effects on the phosphorylation level of ERK in B16F10 melanoma cells when the cells were treated with imperatorin (6.25, 12.5, and 25 μM) for 3 h: (**a**) Western blotting results; (**b**) protein levels of P-ERK/ERK. Β-Actin was used as a loading control. The results are presented as the mean ± SD from three independent measurements using ImageJ software. *** *p* < 0.001 vs. the untreated control group.

Mitogen-activated protein kinases (MAPKs), including extracellular signal-regulated kinase (ERK), p38 and c-Jun N-terminal kinase (JNK), are deeply involved in melanogenesis. In particular, ERK, through its phosphorylation, is responsible for regulating MITF protein stability, i.e., the phosphorylation of MITF decreases its stability and leads to its degradation in proteasomes, and ERK dephosphorylation paradoxically interrupts MITF degradation, thereby activating melanogenesis [35,36,37]. Therefore, we next sought to examine whether imperatorin regulates MITF activity via a MAPK pathway. Western blot analysis showed that treatment with imperatorin significantly decreases ERK phosphorylation in a concentration-dependent manner (Figure 6) but did not have a significant effect on JNK and p38 phosphorylation. These results showed that imperatorin protects MITF from degradation via the phosphorylation of ERK, thereby stimulating melanogenesis. Accordingly, we posit that ERK inactivation is important for imperatorin-induced melanogenesis.

### 2.5. Effects of Imperatorin on the AKT/GSK-3β/β-Catenin Signaling Pathways

**Figure 7:**
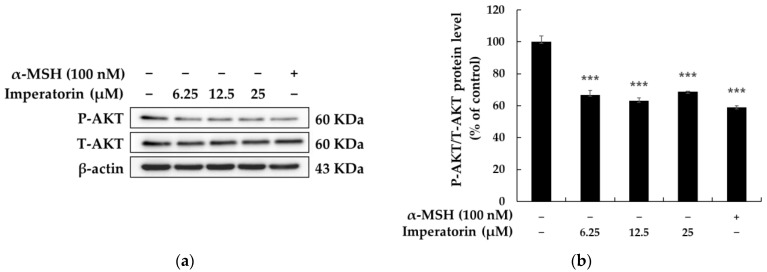
The effects on the protein expression of AKT in B16F10 melanoma cells when the cells were treated with imperatorin (25, 12.5 and 6.25 μM) for 4 h: (**a**) Western blotting results; (**b**) protein levels of MITF. β-Actin was used as a loading control. The results are presented as the mean ± SD from three independent measurements using ImageJ software. *** *p* < 0.001 vs. the untreated control group.

**Figure 8:**
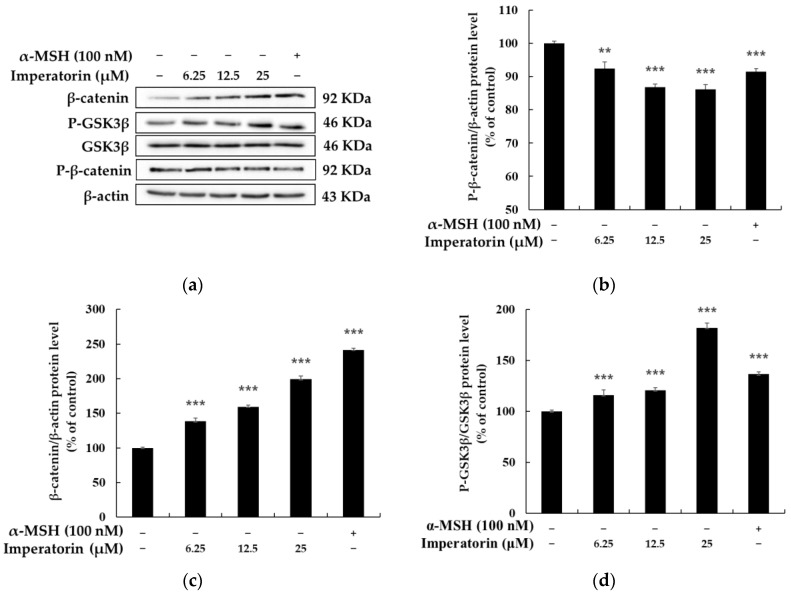
The effects on the protein expression of β-catenin, P-GSK3B, and P-β-catenin in B16F10 melanoma cells when the cells were treated with imperatorin (25, 12.5, and 6.25 μM) for 24 h: (**a**) Western blotting results; protein levels of (**b**) P-β-catenin, (**c**) β-catenin, and (**d**) P-GSK3β. β-Actin was used as a loading control. The results are presented as the mean ± SD from three independent measurements using ImageJ software. ** *p* < 0.01 and *** *p* < 0.001 vs. the untreated control group.

There have recently been numerous reports that the PI3K/AKT (phosphatidylinositol 3-kinases/protein kinase B)/GSK-3β/β-catenin signaling pathways are closely related to melanocyte development and melanogenesis. AKT phosphorylation and activation lead to the phosphorylation of GSK-3β, which results in GSK3β inactivation and the inhibition of β-catenin degradation, which increases its stability. The accumulation of β-catenin in the cytoplasm promotes nuclear translocation, and MITF is transcriptionally upregulated, which eventually leads to induction in the expression of genes involved in melanogenesis [38,39,40]. To evaluate whether imperatorin affects these signaling pathways, we assessed the phosphorylation status of AKT using Western blot analysis. As shown in Figure 7, various concentrations of imperatorin (12.5 and 25 µM) significantly inhibited AKT phosphorylation. We next investigated whether imperatorin induces melanogenesis via the Wnt/β-catenin signaling pathway in B16F10 cells. The results showed that imperatorin increased the levels of P-GSK3β (Ser 9) and β-catenin compared to the untreated control group. However, imperatorin inhibited P-β-catenin expression compared to the untreated control group (Figure 8). These data indicate that the inhibitory effects of imperatorin on melanogenesis are likely to be associated with AKT/GSK-3β/β-catenin signaling pathways.

### 2.6. Effects of Imperatorin on Signaling Pathways by Specific Inhibitors

**Figure 9:**
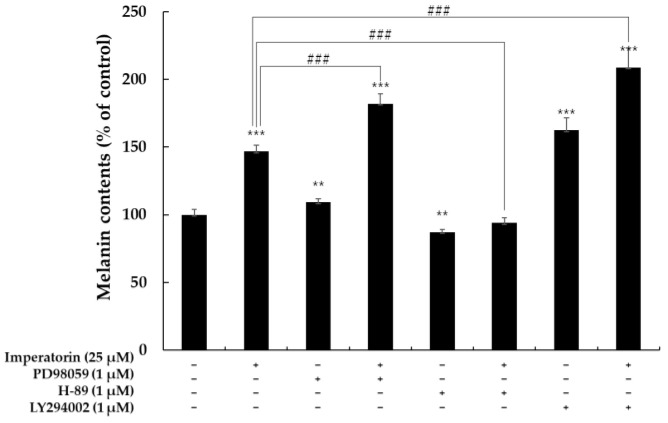
Effect of inhibitor on imperatorin-indued melanin contents in B16F10 cells. To determine the involvement of ERK, AKT, PKA enzyme in melanogenesis, melanin content was conducted using the PD98095, H-89, and LY294002. The results are presented as the mean ± SD from three independent measurements using the Image J. ** *p* < 0.01 and *** *p* < 0.001 vs untreated group. ^###^ *p* < 0.001 vs imperatorin treated group.

To further confirm the role of the signal pathways in imperatorin-induced melanin synthesis, we used the specific inhibitors ERK inhibitor (PD98059), PKA inhibitor (H-89), and AKT inhibitor (LY294002). Cells were pretreated with specific inhibitors 1 h before the addition of imperatorin, then incubated for 72 h for the measurement of melanin content. As shown in Figure 9, imperatorin treatment increased melanin content; these effects were remarkably activated by PD98059 and LY294002. On the other hand, simultaneous treatment of H-89 and Imperatorin slightly increased melanin content than Imperatorin alone. These results indicate that ERK-PKA-AKT-mediated signaling is essential for melanin production increased by imperatorin.

### 2.7. Primary Skin Irritation Test

We then tested the potential of imperatorin in topical application through primary human skin irritation tests. Imperatorin was applied to patches of skin at concentrations of 25 and 50 µM, for up to 24 h. Then, the patches were observed for 20 min and 24 h after the imperatorin was removed. Squalene (solvent) was used as a negative control. As shown in Table 1, the test substance (imperatorin) was classified as causing “no to slight irritation” in terms of primary irritation potential on human skin.

## 3. Discussion

Melanin is a natural biopolymer and an important determinant of human skin color and is known to contribute to different biological processes and to protect organisms from ultraviolet irradiation, detoxification, and embryonic development. Additionally, the inappropriate metabolic processing of melanin, which has been reported to cause skin diseases such as vitiligo and albinism, includes three significant steps: the synthesis, transport, and degradation of melanin. Although many melanogenic stimulators have been developed, they can cause serious side effects, such as allergies, contact dermatitis, eczema, and cytotoxicity. Accordingly, more extensive studies have been conducted on melanin biosynthesis and its specific mechanisms to develop new melanogenesis stimulators using natural products for the treatment of hypopigmentation in diseases such as vitiligo [1,2,3,4,5].

Imperatorin and isoimperatorin are naturally occurring active furocoumarin products that are found in the fruits of *Citrus* species and some species of *Angelica* used as medicines. Previously, Zeng et al. [41] reported that isoimperatorin reduces melanin content in keratinocytes via miR-3619/CSTB and miR-3619/CSTD axes, which supports the proposal that isoimperatorin functions as a melanogenic inhibitor. Additionally, Cho et al. [42] reported that isoimperatorin and imperatorin, of various active compounds isolated from *Angelica dahurica*, significantly inhibit tyrosinase synthesis in B16 melanoma cells. However, as shown in Figure 2, we instead found in our study on the associations between imperatorin derivatives and melanogenesis that they increased melanin biosynthesis; i.e., although the interesting contradictory results for imperatorin and isoimperatorin require further experiments for confirmation, such as through experiments on in vitro human epidermal melanocytes and in vivo human subjects, we found that imperatorin and isoimperatorin stimulated melanogenesis and characterized the mechanisms involved in this activation.

Mouse melanoma B16F10 cells share most similar melanogenic mechanisms with human epidermal melanocytes and, thus, are commonly used in in vitro assays of melanin synthesis [43]. Therefore, in this study, we investigated the effects of imperatorin and isoimperatorin on melanin biosynthesis and its molecular mechanisms in B16F10 cells to understand the underlying signaling pathways.

The current study demonstrated that within the safe concentration range (6.25–25 μM), imperatorin and isoimperatorin enhanced melanin synthesis by activating the rate-limiting enzyme tyrosinase without having any cytotoxic effects on the B16F10 cells (Figure 2). Because no significant differences in melanin content and intracellular tyrosinase activity were observed between imperatorin and isoimperatorin concentrations of 25 μM, further experiments were performed to explore the potential molecular mechanisms of imperatorin. To further understand the molecular mechanisms of imperatorin that act on melanogenesis in B16F10 cells, we examined the protein expression levels of important pathways.

Since the most common pigmentation disorder is associated with hypo-expression of MITF, recent research has focused on developing better approaches for its upregulation in treating MITF-mediated melanin deficiency. Therefore, we evaluated the influence of imperatorin on melanogenesis-related enzymes to understand the melanogenesis stimulation mechanisms of imperatorin in detail. Melanogenesis is directly regulated by the major enzymes TRY, TYRP-1, and TYRP-2 [44,45]. In this study, imperatorin significantly increased the expression of TRY, TYRP-1, and TYRP-2, which is consistent with the results observed for melanin synthesis. Although melanin biosynthesis is a complex process, increasing evidence shows that MITF is a critical factor for the pigmentation process in melanocytes. Importantly, MITF regulates the expression of TYR, TYRP-1, and TYRP-2 during melanin production. Our data show that treatment with imperatorin enhances the expression of MITF, thereby leading to an increase in intracellular TYR activity and melanin content in mouse B16F10 cells. These observations suggest that imperatorin augments the functionality of MITF signaling-dependent melanin biosynthesis in B16F10 cells.

cAMP is considered a major signaling molecule that regulates pigmentation by activating PKA-mediated CREB phosphorylation via the binding of α-MSH to MC1R. Thus, natural products that target the cAMP/PKA/CREB axis act as potent regulators of melanogenesis. In addition, CREB increases melanogenesis by activating MITF expression, which promotes the transcription of TYR, TYRP-1, and TYRP-2 [31,32,33,34]. Chen et al. [46] previously reported that knockout of the MITF-M gene caused albinism in mice, which confirmed the importance of MITF as a key transcription factor in melanin production. In addition, small interfering RNA-mediated MITF silencing in melanoma cells significantly reduces melanin content by inhibiting TYR, TYRP-1, and MC1R, which indicates that the transcriptional activation of MITF is a promising strategy for treating hypopigmentation [47]. In this study, imperatorin was identified as a potent activator of PKA and CREB expression, which suggests that the cAMP/PKA/CREB/MITF axis could be responsible for the melanogenic properties of imperatorin.

In addition to PKA/CREB signaling, the MAPK family (which includes ERK, JNK and p38) is known to play an important role in the regulation of melanin production. The activation of ERK is associated with a reduction in MITF degradation and pigmentation, while the inhibition of ERK increases melanin synthesis. Thus, MAPK signals in melanocytes are promising targets for the development of cosmetics and treatments for skin pigmentation through which MITF expression and stability can be increased [35,36,37]. Therefore, we evaluated the effectiveness of imperatorin for the inhibition of the ERK signaling pathway to further explore the molecular mechanisms of melanin synthesis in B16F10 cells. As shown in Figure 6, imperatorin reduced the ERK phosphorylation concentration in a concentration-dependent manner. Therefore, it could be concluded that the inhibition of ERK phosphorylation by imperatorin is one of the basic mechanisms that induced pigmentation.

Another important signaling pathway that involves PI3K/AKT/GSK3β has been found to regulate the transcriptional activity of MITF. In fact, α-MSH-induced cAMP can inhibit AKT activity by inhibiting the phosphorylation of AKT in Thr308 and Ser473 via a PI3K-dependent mechanism. Thus, non-phosphorylated GSK3β, which is a downstream molecule of AKT, activates and phosphorylates MITF in Ser289 and upregulates the tyrosinase family genes, which results in melanin production. For example, the inhibition of phosphatidylinositol 3-kinase (PI3K) and downstream AKT signaling increases tyrosinase expression and melanin content, whereas the constitutive activation of AKT decreases melanin content. In this study, imperatorin decreased AKT activity and AKT phosphorylation in Thr308 and Ser473, followed by the inhibition of GSK3β phosphorylation. Among other effects, this GSK3β activation led to the activation of MITF and the increased expression of tyrosinase.

The relationship between Wnt/β-catenin signaling and melanocyte differentiation has been demonstrated by research showing that β-catenin accumulates after Wnt/β-catenin signaling activation and forms a complex with lymphocyte enhancer factor-1 that upregulates the expression of MITF genes. In addition, β-catenin can interact directly with the MITF protein itself, which can then activate downstream MITF-specific target genes. According to a recent study, the activation of the cAMP/PKA pathway promotes the phosphorylation of β-catenin in Ser675 and the phosphorylation of β-catenin in Ser9, which inhibits the degradation of β-catenin and the translocation of β-catenin to the nucleus, thereby promoting the transcription of Wnt/β-catenin target genes. Our results showed that imperatorin could activate the expression of β-catenin and MITF, which in turn upregulated the induction of tyrosinase, TYRP-1 and TYRP-2 and melanin production. In addition, imperatorin upregulates the expression of p-GSK3β (Ser9), which suggests that imperatorin could mediate melanogenesis by activating the Wnt/β-catenin pathway via activation of the cAMP/PKA pathway [38,39,40].

Finally, we tested whether imperatorin could potentially be applied as a topical material using primary human skin irritation tests. To determine whether 25 or 50 μM imperatorin had stimulation or sensation potential, these concentrations were tested on the normal skin (upper back) of 31 volunteers. In this analysis, imperatorin was judged as causing “no to slight irritation”.

**Figure 10:**
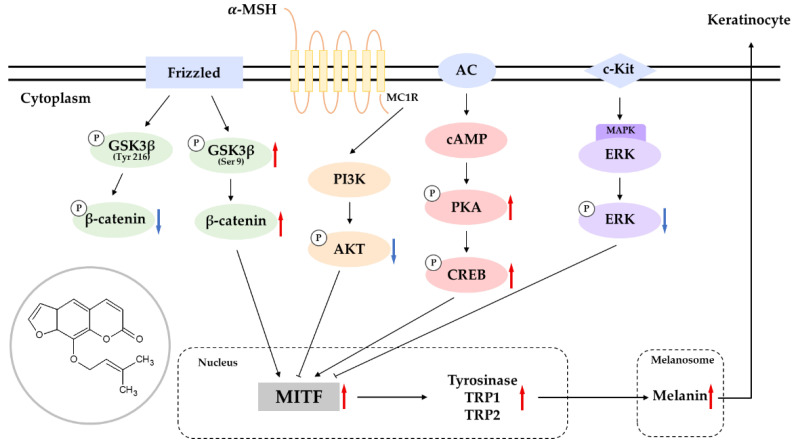
Schematic diagram of the proposed mechanism regulating the stimulative action of imperatorin on melanogenesis.

When taken together, these data show that imperatorin induces melanogenesis by inducing the expression of MITF, which is an important protein that is involved in the expression of other melanogenetic proteins, including TRY and TYRPs. In addition, we found that the imperatorin-induced expression of MITF is dependent on the activation of signaling pathways involving ERK, AKT, PKA/CREB, and Wnt/β-catenin (Figure 10). These results suggest that imperatorin could prevent the pathogenesis of pigmentation disorders when used as a topical agent. The possible involvement of the above signaling pathways in stimulating melanin synthesis through imperatorin still needs to be investigated in the future. Although we have shown the melanogenic effects of imperatorin in B16F10 melanoma cells, the relative effectiveness of imperatorin in human normal melanocytes remains to be determined in future study [48,49]. In addition, the efficacy and safety of imperatorin-induced melanogenesis stimulation must be evaluated in vitiligo animal models and humans.

## 4. Materials and Methods

### 4.1. Chemicals and Reagents

The isoimperatorin (≥98%) was purchased from Cayman Chemical Company (Ann Arbor, MI, USA), and the imperatorin (≥98%) was purchased from Tokyo Chemical Industry (Chuo-ku, Tokyo, Japan). The reagents 3-(4,5-dimethyl-thiazol-2-yl)-2,5-diphenyl tetrazolium bromide (MTT), L-DOPA, and α-melanocyte stimulating hormone (α-MSH) were purchased from Sigma-Aldrich (St. Louis, MO, USA). Dimethyl sulfoxide (DMSO) and phosphate-buffered saline (PBS) were purchased from Biosesang (Seongnam-si, Gyeonggi-do, Korea). Dulbecco’s modified Eagle’s medium (DMEM) and penicillin/streptomycin were purchased from Thermo Fisher Scientific (Waltham, MA, USA). The primary antibodies for TYR, TYRP-1, TYRP-2, CREB, and p-CREB were obtained from Santa Cruz Biotechnology Inc. (Santa Cruz, CA, USA). The p-ERK, p-GSK3B, P-β-catenin, p-PKA, and p-AKT were purchased from Cell Signaling Technology Inc. (Beverly, MA, USA). The secondary antibodies (anti-mouse IgG and anti-rabbit IgG) were also purchased from Cell Signaling Technology Inc. (Beverly, MA, USA).

### 4.2. Cell Cultures

B16F10 melanoma cells were obtained from the Korean Cell Line Bank (Seoul, Korea). The cells were cultured in DMEM, which was supplemented with 10% FBS and 1% antibiotics and maintained at 37 °C in a humidified incubator that was supplemented with 5% CO_2_.

### 4.3. Cell Viability

Cell cytotoxicity was determined via MTT assays. The B16F10 melanoma cells (1 × 10^4^ cells/well) were seeded in each well of 24-well plates and cultured in an incubator for 24 h. Isoimperatorin and imperatorin were diluted to various concentrations and added into each well and incubated for 3 days. An MTT reagent (0.5 mg/mL) was added to the cells for 3 h at 37 °C. The medium was subsequently removed. The insoluble MTT formazan crystals were solubilized using DMSO. The absorbance at 560 nm was measured using a microplate spectrophotometer (Tecan, Mannedorf, Switzerland). Cell viability was measured by comparing the percentage of cell viability of the treated cells to the 100% viability of the untreated cells (control group).

### 4.4. Determination of Melanin Content

B16F10 melanoma cells (7.0 × 10^4^ cells/well) were seeded into 60 mm cell culture dishes. After 24 h of incubation, the cells were treated with various concentrations of the samples or 100 nM of α-MSH (a-melanocyte stimulating hormone) in the DMEM. After incubation, the cells were harvested and washed twice with PBS and the intercellular melanin was solubilized in 1 N of NaOH at 80 °C for 1 h. The aliquots of the cell lysates were transferred to 96-well culture plates, and the absorbance of melanin contents were estimated using an ELISA microplate spectrophotometer (Tecan, Mannedorf, Switzerland) at 405 nm. The melanin contents were calculated as percentages of change compared to the untreated cells.

### 4.5. Measurement of Cellular Tyrosinase Activity

B16F10 melanoma cells were seeded in the same plates as for the melanin content analysis and treated with various concentrations of the samples or α-MSH (100 nM). They were then removed from the medium in each dish and processed in an RIPA buffer (a sterile solution of 150 mM of sodium chloride, 1% Triton X-100, 1% sodium deoxycholate, 0.1% SDS, 50 mM of Tris–HCl, pH 7.5 and 2 mM of EDTA) for 20 min, which also contained a 1% protease inhibitor cocktail. The cells were scraped and collected in e-tubes. A supernatant liquid was prepared by centrifuging the mixture at 15,000 rpm for 20 min at −8 °C. The protein concentration of the supernatant liquid was quantified using a BCA kit. Then, 20 μL of the quantified protein solution was added to a 96-well plate and 80 μL of L-DOPA (2 mg/mL) was dissolved in a sodium phosphate buffer and was treated in each well. The reaction was carried out at 37 °C for 30 min and then measured using an ELISA microplate spectrophotometer at 490 nm.

### 4.6. Western Blot Analysis

B16F10 melanoma cells (7.0 × 10^4^ cells/well) were seeded into 60 mm cell culture dishes. The cells were treated with imperatorin (25, 12.5, and 6.25 μM) and α-MSH (100 nM) for each protein expression timescale. After treatment, the medium in each dish was removed and washed twice with cold PBS. A lysis buffer that contained a 1% protease inhibitor cocktail was reacted for 20 min. The cells were collected in e-tubes. The cell-dissolved solvent was then centrifuged at 15,000 rpm for 20 min at -8 °C and the protein was quantified using a BCA kit with a supernatant liquid. The cell dissolution was mixed with a 2× Laemmli sample buffer and heated at 100 °C for 5 min. Each sample was loaded with 10% sodium dodecyl sulfate (SDS) polyacrylamide gels and transferred to PVDF membranes. The membranes were blocked with 5% skim milk for 2 h, washed six times for 10 min over 1 h using 1× TBS-T, and then treated with the primary antibodies overnight. After that, the membranes were washed six more times for 10 min each. Then, the secondary antibodies were treated at room temperature for 2 h. Again, the membranes were washed six times with TBST and reacted with an ECL kit. For our quantitative analysis, the amount of protein expression was determined using ImageJ software ver. 1.4 (NIH, Bethesda, MD, USA).

### 4.7. Primary Skin Irritation Test

Overall, 31 volunteers (healthy females) aged between 20 and 60 years and had never experienced irritant and/or allergic contact dermatitis were included in this study. Their ages ranged from 29 to 53 years, with a mean age of 43.19 ± 5.97 years. Squalene-added imperatorin was prepared as a negative control and applied at a concentration of 100 μM. Primary skin irritation responses were assessed according to the PCPC guidelines. The reaction results for each test substance were calculated from the formula shown below [50,51]. The study was approved by the Industrial Review Board (IRB) of Dermapro Inc. and was conducted in accordance with the Helsinki Declaration as a declaration of the ethical principles for medical research after obtaining written consent from each volunteer (IRB number: 1-220777-A-N-01-DICN22080).

$$\mathrm{Response}=\frac{\sum \left(\mathrm{Grade}\times \mathrm{No}.\;\mathrm{of}\;\mathrm{Responders}\right)}{4\;\left(\mathrm{Maximum}\;\mathrm{Grade}\right)\times n\;\left(\mathrm{Total}\;\mathrm{Subjects}\right)}\times 100\times 1/2$$

### 4.8. Statistical Analysis

All the experimental results are expressed as the mean ± the standard deviation (SD) of at least three independent experiments. Statistical analyses were performed using Student’s t-tests or one-way ANOVA tests using IBM SPSS v. 20 (SPSS Inc., Armonk, NY, USA). Additionally, *p*-values < 0.05 (*) or 0.01 (**) were marked as statistically significant.

## Figures and Tables

**Table 1:** The results from the primary human skin irritation tests (*n* = 31).

No.	Test Sample	No. of Respondents	20 min after Removal				24 h after Removal				Reaction Grade (R) *		
			+1	+2	+3	+4	+1	+2	+3	+4	24 h	48 h	Mean
1	Imperatorin (25 μM)	2	-	-	-	-	2	-	-	-	0	2	0.8
2	Imperatorin (50 μM)	1	-	-	-	-	1	-	-	-	0	1	0.4
3	Squalene	0	-	-	-	-	-	-	-	-	0	0	0

The reactions were assessed 20 min and 24 h after the removal of the treatment by the investigator, according to PCPC guidelines (2014). * The range of irritation from “no to slight irritation”: 0.00 ≤ R < 0.87.

## Data Availability

Not applicable.
